# Novel 3D light microscopic analysis of IUGR placentas points to a morphological correlate of compensated ischemic placental disease in humans

**DOI:** 10.1038/srep24004

**Published:** 2016-04-05

**Authors:** Eva Haeussner, Christoph Schmitz, Hans-Georg Frank, Franz Edler von Koch

**Affiliations:** 1 Department of Anatomy II, LMU Munich, Pettenkoferstr. 11, 80336 Munich, Germany; 2 Clinic for Obstetrics and Gynecology Dritter Orden, Menzinger Str. 44, 80638 Munich, Germany

## Abstract

The villous tree of the human placenta is a complex three-dimensional (3D) structure with branches and nodes at the feto-maternal border in the key area of gas and nutrient exchange. Recently we introduced a novel, computer-assisted 3D light microscopic method that enables 3D topological analysis of branching patterns of the human placental villous tree. In the present study we applied this novel method to the 3D architecture of peripheral villous trees of placentas from patients with intrauterine growth retardation (IUGR placentas), a severe obstetric syndrome. We found that the mean branching angle of branches in terminal positions of the villous trees was significantly different statistically between IUGR placentas and clinically normal placentas. Furthermore, the mean tortuosity of branches of villous trees in directly preterminal positions was significantly different statistically between IUGR placentas and clinically normal placentas. We show that these differences can be interpreted as consequences of morphological adaptation of villous trees between IUGR placentas and clinically normal placentas, and may have important consequences for the understanding of the morphological correlates of the efficiency of the placental villous tree and their influence on fetal development.

The villous tree of the human placenta enables feto-maternal exchange. The exchange function of the villous tree is mainly controlled by the trophoblast, an epithelium with an apical, syncytialized layer^1^,^2^,^3^,^4^. The trophoblast is positioned at the surface of a multi-branched villous tree structure which consists of stromal cells and vessels. The villous tree is a three-dimensional (3D) structure with characteristic branching pattern and multiple branching points (nodes), and can be analyzed by tree-specific morphometric assessments similar to those established for other naturally occurring tree structures^5^. Variations in the architecture of branches and nodes of the villous trees have been in focus of microscopic placental research for decades^3^,^4^. It has been hypothesized that structural aberrances of the angiogenetically driven branching of villous trees are an essential correlate of pathologic course of pregnancies^6^,^7^. A frequent^4^,^8^ and severe pregnancy disease is intrauterine growth retardation (IUGR;^1^,^4^,^9^) which is an important cause of iatrogenic preterm birth and increases life-long health risks for the child^10^. Numerous histological findings (infarction, immaturity, hypermaturity, hyperangiogenesis, etc.^4^,^6^,^11^) were documented for IUGR with conventional, two-dimensional (2D) histology (i.e., by analyzing thin sections of human placentas) but could so far not be consistently translated into quantitative 3D morphological topology of the villous trees.

The 3D topology of the villous tree cannot reliably be analyzed on thin (2D) histological sections because the identification of villi as respectively terminal, intermediate or small peripheral stem villi is not unequivocally possible^12^. Furthermore, branching hierarchy, nodes and parameters like branching angles, tortuosity and branch length cannot be determined on thin (2D) histological sections at all^12^,^13^.

Recently, we introduced a novel light microscopic method for accurate 3D tracing, reconstruction, visualization and quantitative analysis of the topological position of branches and nodes of human placental villous trees^12^,^13^. This method allows instant determination of topological parameters such as hierarchy of branches, branching angles and length of branches of isolated peripheral villous trees. The principles of this novel method are summarized in Fig. 1. When applied to the analysis of a population of clinically normal placentas this novel method revealed a correlation between the feto-placental weight ratio at birth and villous branching angles of branches in terminal positions^13^.

In the present study we hypothesized that such advanced 3D microscopic analysis will reveal differences of e.g. branching and length and/or tortuosity of branches in defined topological positions of isolated peripheral villous trees of IUGR placentas as compared to villous trees of clinically normal placentas.

We tested this hypothesis on two collections of human placentas, i.e., n = 40 IUGR placentas and n = 50 clinically normal placentas. Under careful dissection we prepared and isolated peripheral parts of villous trees and analyzed these tissue samples with the software, Neurolucida (MBF Bioscience, Williston, VT, USA). We used settings adapted to the requirements of villous branching^13^.

We found that individual mean branching angles of villi in terminal position (i.e., one value per investigated placenta) and individual mean tortuosity of villi in directly preterminal position were significantly different statistically between IUGR placentas and clinically normal placentas.

## Results

### Macroscopic analysis for sample validation

No statistically significant differences between the IUGR placentas and the clinically normal placentas were found in the mean feto-placental weight ratio (i.e., the ratio between placental weight and fetal weight at birth) and the mean roundness of the outer placental rim (Table S1 in Supplementary Information). All other investigated parameters (gestational age, birth weight, placental weight, surface area of the placenta, thickness of the placenta, longest diameter of the placental disk and shortest diameter of the placental disk) were on average significantly smaller statistically in the IUGR placentas compared to the clinically normal placentas (Table S1 in Supplementary Information). These data are in line with results of numerous earlier studies^14^,^15^,^16^,^17^ and, thus, validate the present collections of placentas for the purpose of research on IUGR placentas.

### Microscopic analysis and branch topology

**Figure 1:**
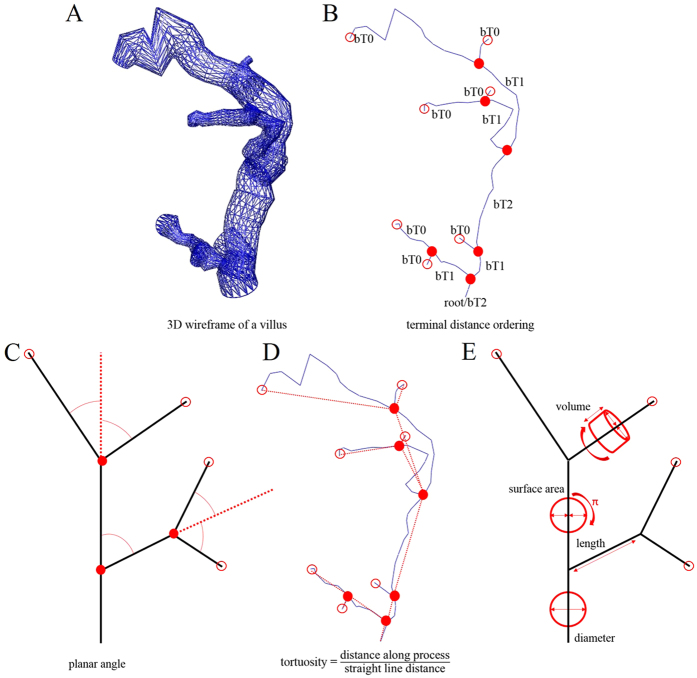
Principles of our novel light microscopic method for accurate 3D microscopic tracing, reconstruction, visualization and quantitative analysis of the topological position of branches and nodes of human placental villous trees. (**A,B**) two graphs of the same villous tree, obtained by 3D microscopic tracing and reconstruction. The surface of the villous tree is shown as a rendered 3D wireframe (**A**), whereas (**B**) shows the skeletonized view of the villous tree. The terminal distance order is given for each branch (abbreviated bT). The number behind bT indicates the number of nodes (filled red dots) to the nearest terminal end of the tree. Accordingly, bT0 indicates villous branches in terminal position of the villous tree, bT1 villous branches in direct preterminal position of the villous tree, and bT2 villous branches that are two nodes apart of the next terminal end. The root gives the start of villous tracing. (**C**) sketch of a human placental villous tree showing connections between branching nodes (filled red dots) and/or terminal ends (open dots). The planar branching angle of a given branch (black lines) was defined as the change in the direction of the branch with respect to the direction of the branch at the next higher order (red dotted line). The direction of each branch was derived from its endpoint (i.e., a terminal end in case of bT0 or a branching node in case of bT1 and bT2). (**D**) the same skeletonized view of a human placental villous tree as shown in (**B**), indicating tortuosity of individual branches. Tortuosity is a measure of how twisted a branch is, and is defined as the ratio between the actual distance in space along a branch (skeleton lines) and the shortest distance in space between the corresponding branching nodes and/or terminal ends (red lines). Thus, a straight line has a tortuosity of 1, and a branch with twists and turns a tortuosity of greater than 1 42,43,44,45. (**E**) the same sketch of a human placental villous tree as shown in (**C**), indicating measurement of the parameters diameter, length, surface area and volume.

**Figure 2:**
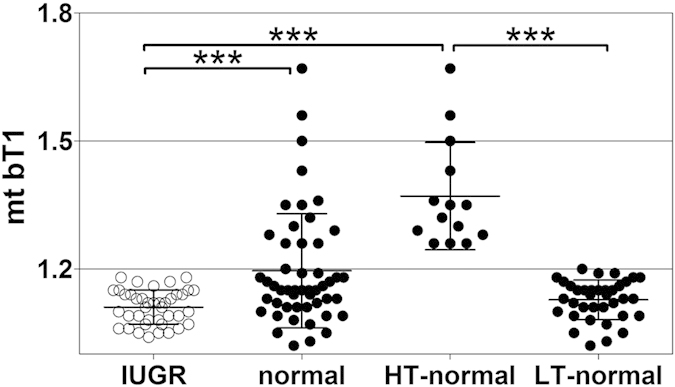
Individual mean tortuosity of villous branches in directly preterminal position of IUGR placentas and clinically normal placentas. The panel shows mean, standard deviation and single values (one per placenta) of the individual mean tortuosity of the branches in directly preterminal position (mt bT1) in IUGR placentas and clinically normal placentas, with the latter split into placentas with either high (HT-normal) or low (LT-normal) tortuosity (details are provided in the main text). The term bT1 is defined in Fig. 1B. Results of statistical analysis (post-hoc Dunn’s test for pairwise comparisons after Kruskal-Wallis test; p < 0.001) are indicated. *p < 0.05; **p < 0.01; ***p < 0.001.

The individual mean tortuosity of villous branches in directly preterminal position of the villous tree (i.e., at position bT1 according to our new classification^13^; one mean value per investigated placenta) was significantly lower statistically in the IUGR placentas than the clinically normal placentas (p = 0.0002; Fig. 2). Detailed analysis revealed that the observed difference between the IUGR placentas and the clinically normal placentas was caused by a subset of clinically normal placentas with individual mean tortuosity of villous branches at position bT1 of more than 1.2, i.e., a higher value than found for any of the investigated IUGR placentas. We therefore divided the clinically normal placentas into two subgroups, i.e., placentas with individual mean tortuosity of villous branches in bT1 of more than 1.2 (henceforth referred to as high tortuosity at position bT1; HT-normal) or less than 1.2 (henceforth referred to as low tortuosity at position bT1; LT-normal), respectively. Accordingly, comparison between the IUGR placentas, the HT-normal placentas and the LT-normal placentas showed statistically significant differences in mean values of the individual mean tortuosity of villous branches at position bT1 between the IUGR placentas and the HT-normal placentas as well as between the HT-normal placentas and the LT-normal placentas, but not between the IUGR placentas and the LT-normal placentas (Fig. 1).

**Figure 3:**
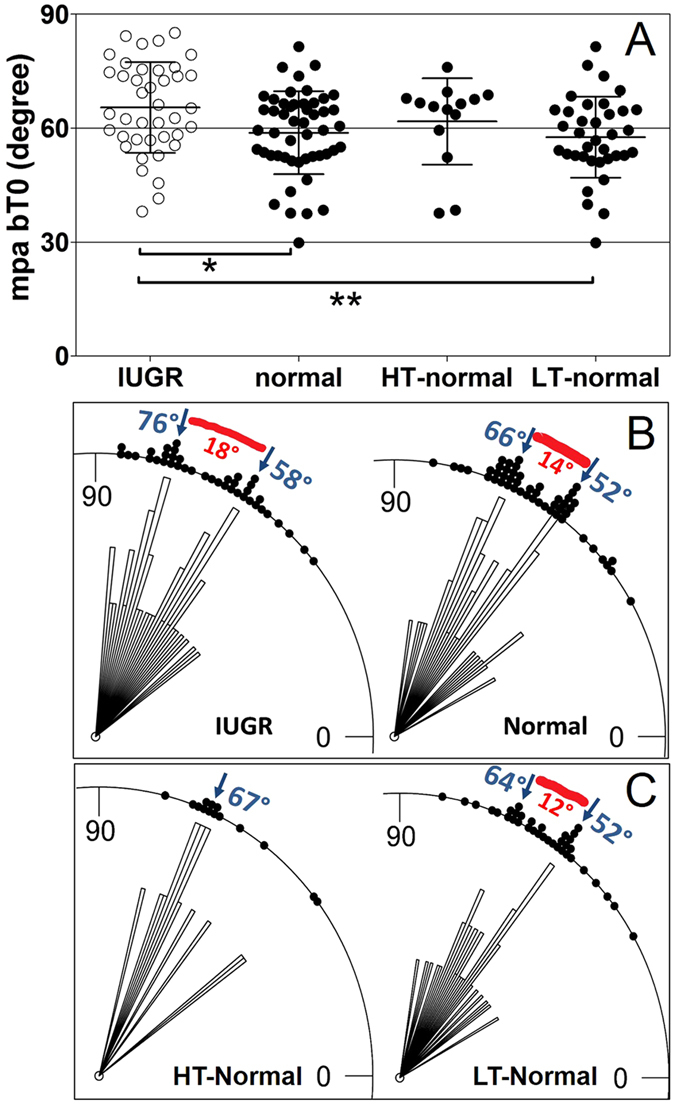
Individual mean planar angles of villous branches in terminal position in IUGR placentas and clinically normal placentas. (**A**), mean, standard deviation and single values (one per placenta) of the individual mean planar angle of the branches in terminal position (mpa bT0) in IUGR placentas and clinically normal placentas, with the latter split into placentas with either high (HT-normal) or low (LT-normal) tortuosity. The term bT0 is defined in Fig. 1. Results of statistical analysis (post-hoc Dunn’s test for pairwise comparisons after Kruskal-Wallis test; p < 0.0132) are indicated. *p < 0.05; **p < 0.01; ***p < 0.001. (**B,C**) rose diagrams of single values (one per placenta) of the individual mean planar angle of the branches in terminal position (mpa bT0) in IUGR placentas and clinically normal placentas (**B**), with the latter split into placentas with either high (HT-normal) or low (LT-normal) tortuosity (**C**). Values that occurred most frequently are labelled with blue arrows. The distance between these maxima is indicated by red segments.

The individual mean planar branching angle of villous branches in terminal position of the villous tree (i.e., at position bT0 according to our new classification^13^; one value per investigated placenta) of the IUGR placentas was significantly larger statistically than in clinically normal placentas (p = 0.007; Fig. 3A). When comparing the IUGR placentas with the HT-normal placentas and the LT-normal placentas, the difference between the IUGR placentas and the LT-normal placentas was statistically significant, but neither the difference between the IUGR placentas and the HT-normal placentas nor between the HT-normal placentas and the LT-normal placentas (Fig. 3A). Rose diagrams showed that the distributions of the individual mean planar branching angle of villous branches at position bT0 had two maxima in case of the IUGR placentas (found at 58 and 76 degrees; Fig. 3B), the clinically normal placentas (52 and 66 degrees; Fig. 3B) and the LT-normal placentas (52 and 64 degrees; Fig. 3C). Hartigan´s Dip test confirmed non-unimodality of these data. In contrast, the distribution of the individual mean planar branching angle of villous branches at position bT0 of the HT-normal placentas had only one maximum at 67 degree (Fig. 3C).

**Figure 4:**
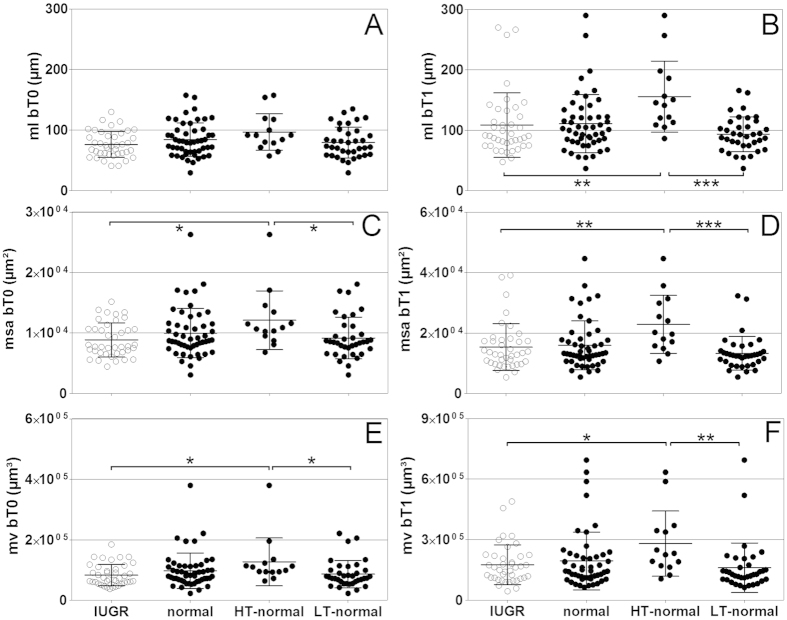
Individual mean length, surface area and volume of villous branches in terminal and directly preterminal position of IUGR placentas and clinically normal placentas. The panels show mean, standard deviation and single values (one per placenta) of the mean length (ml) of the branches at terminal position bT0 (A; ml bT0; result of Kruskal-Wallis test: p = 0.123) and directly preterminal position bT1 (B; ml bT1; p = 0.001), of the mean surface area (msa) of the branches at positions bT0 (C; msa bT0; p = 0.027) and bT1 (D; msa bT1; p = 0.002), and of the mean volume (mv) of the branches in bT0 (E; mv bT0; p = 0.028) and bT1 (F; mv bT1; p = 0.008) in IUGR placentas and clinically normal placentas, with the latter split into placentas with either high (HT-normal) or low (LT-normal) tortuosity in bT1 as defined in Fig. 2. The terms bT0 and bT1 are defined in Fig. 1B. Results of statistical analysis (post-hoc Dunn’s tests for pairwise comparisons after Kruskal-Wallis test with p < 0.05) are indicated. *p < 0.05; **p < 0.01; ***p < 0.001.

We found no statistically significant difference in the mean values of the individual mean length of the villous branches at position bT0 between the IUGR placentas, the clinically normal placentas, the HT-normal placentas and the LT-normal placentas (Fig. 4A). Furthermore, the IUGR placentas were not significantly different statistically from the clinically normal placentas with regard to the mean values of the individual mean length of the villous branches at position bT0 (Fig. 4B), the individual mean surface areas of the villous branches at positions bT0 (Fig. 4C) and bT1 (Fig. 4D), and the individual mean volume of the villous branches at positions bT0 (Fig. 4E) and bT1 (Fig. 4F). Furthermore, for none of these parameters statistically significant differences were found between the IUGR placentas and the LT-normal placentas (Fig. 4B–F). In contrast, the HT-normal placentas showed statistically significantly higher mean values for all these parameters than the IUGR placentas and the LT-normal placentas (Fig. 4B–F).

Birth weight, placenta weight and the fetoplacental weight ratio were not significantly different statistically between the LT-normal and HT-normal placentas.

## Discussion

**Figure 5:**
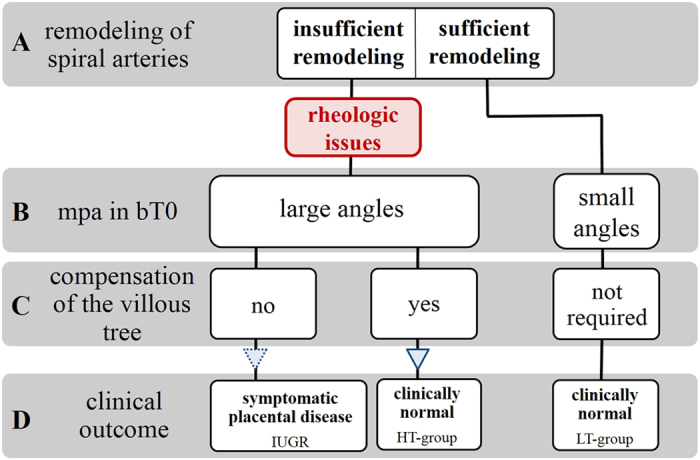
Scheme illustrating our novel hypothesis on pathophysiological interpretation of branching angles and tortuosity of villous branches in positions bT0 and bT1 of human placentas. The proposal starts with the widely accepted early origin of IUGR as insufficient remodeling of uterine spiral arteries (A). Due to rheologic disturbance caused by this insufficient remodeling of uterine spiral arteries, branching angles (mpa) in position bT0 become larger (B; c.f. Fig. 6). For reasons that are currently unknown some placentas cannot compensate this rheologic disturbance and develop into IUGR placentas. In contrast, other placentas with insufficient remodeling of uterine spiral arteries (and, thus, rheologic disturbance and large branching angles at position bT0) develop highly tortuous branches in position bT1 as morphological correlate of successful compensation of rheologic disturbance (C). As a result this subset of placentas (i) shares some morphological characteristics with IUGR placentas (i.e., large branching angles in position bT0; c.f. Fig. 3) and (ii) shows certain morphological characteristics that were neither found in IUGR placentas nor in placentas with sufficient remodeling of uterine spiral arteries (i.e., higher mean tortuosity of the branches in position bT1, higher length of the branches in position bT1 as well as higher mean surface area and higher mean volume of the branches in positions bT0 and bT; c.f. Fig. 4). Because of this successful compensation of rheologic disturbance these placentas present as clinically normal, but are morphologically distinct from other clinically normal placentas in which no insufficient remodeling of uterine spiral arteries occurred.

**Figure 6:**
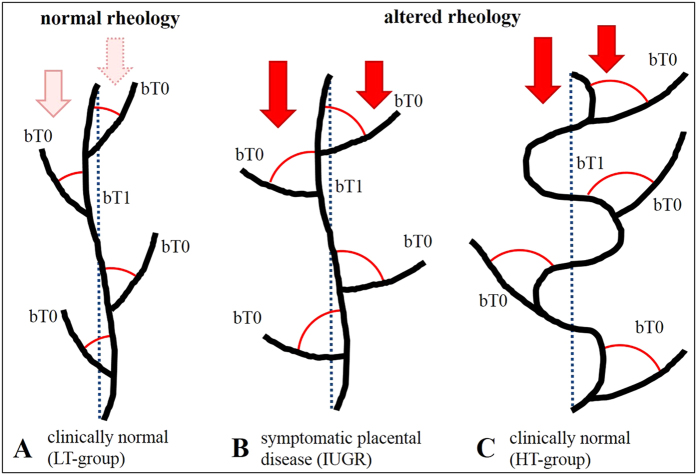
Scheme of hypothesized reactions of the placental villous tree to altered rheology in the intervillous space. (**A**) without altered rheology in the intervillous space branching angles of villi in position bT0 and tortuosity of villi in position bT1 are small. These placentas present as clinically normal. (**B**) altered rheologic properties of the blood flow in the intervillous space are indicated by red arrows. The branching angles of branches in position bT0 increase (red circular arcs). Without compensation these placentas develop into IUGR placentas. (**C**) in a subgroup of placentas exposed to altered rheologic properties of the blood flow in the intervillous space highly tortuous (and, thus, longer) branches in position bT1 appear (tortuosity indicated by increased deviation from the straight blue dotted line; not to scale). These placentas develop into clinically normal placentas (**C**).

The method used in the present study is a novel and innovative approach to analyze the topology of the most peripheral parts of the villous tree in 3D^13^. The most relevant finding of the present study was the statistically significant upward shift of mean planar branching angles of villous branches in terminal position bT0 in IUGR placentas compared to clinically normal placentas (Fig. 3). This result can be interpreted in view of earlier findings that in clinically normal placentas, the mean planar branching angle of branches in position bT0 positively correlated with the feto-placental weight ratio^13^. This implies that clinically normal placentas with relatively more placental tissue to nourish the fetus tend to have larger branching angles in position bT0. Overall, the upwards shift of angles in position bT0 can be interpreted as a reaction of the placenta to conditions which are present in IUGR, but also in a subpopulation of clinically normal placentas. This novel concept is visualized in Figs 5 and 6 and explained in the following.

The main pathogenetic mechanism associated with IUGR (and other obstetric syndromes like preeclampsia) is insufficient remodeling of uterine spiral arteries by invading trophoblast in early pregnancy (Fig. 5A)^11^,^18^,^19^,^20^,^21^,^22^. Recently, obstetric syndromes associated with this common early pathogenetic event were summarized in a unifying concept named “ischemic placental disease^18^”. As placentation proceeds to full hematotrophic nutrition beyond the 12^th^ week, insufficient remodeling of uterine spiral arteries is thought to cause rheologic disturbance (i.e., blood flow disturbances) in the intervillous space and at the surface of the villi (Fig. 5B)^22^. Higher branching angles in position bT0 can thus potentially be interpreted as a reaction of the constantly growing villous tree to modified intervillous blood flow (Figs 5C and 6B,C). Such reaction of villous branching to modified blood flow environment could be initiated by direct mechanical force on the villous tree, driving branching into higher angles (Fig. 6B). However, data in favor of such a directly flow-mechanical impact have not yet been published. Another option would be that the modified blood flow environment is recognized by mechanotransduction and signaled to angiogeneically active cells inside the villi. Angiogenesis (especially non-branching angiogenesis in the late phase of pregnancy) is thought to be the main mechanism driving villous branching^4^. Differences in branching angles can thus be interpreted as signs of differences of villous angiogenesis. Indeed, branching angles in vascular beds can be influenced by the mechanism of angiogenesis. An angiogeneic mechanism which is known to modify vascular branching angles (and thus possibly also villous branching as consequence of angiogeneic branching) is intussusceptive angiogenesis^23^,^24^,^25^. This type of angiogenesis modifies branching angles and is also known to be a fast-response mechanism to mechanical stress^26^,^27^. Intussusceptive angiogenesis would be able to respond to e.g. modifications of rheology in the intervillous space. Thus far, to our knowledge, data on the occurrence and relevance of intussusceptive angiogenesis in villous trees of complicated pregnancies are not available.

With the data currently available, it cannot be clarified whether the observations of the present study on branching angles in position bT0 in IUGR placentas and HT-normal placentas reveal a reactive symptom or (in case of IUGR) a cause of growth retardation (Figs 5 and 6). Since the early invasive processes leading to remodeling of uterine spiral arteries are very specific to the human species^22^, animal models with a different type of early placentation and particularly the absence of villous trees are in principle not helpful to move this field forward.

The present study shows for the first time that tortuosity of branches in the directly preterminal position bT1 significantly differs statistically between IUGR placentas and clinically normal placentas (Fig. 2; note that tortuosity as defined in the present study is not identical to the increased formation of knots and syncytial folds at the villous surface which were reported in earlier studies on severe early onset IUGR placentas^11^).

The changes of tortuosity concerned the branch generations in position bT1 and thus the peripheral region of placental villous trees which is considered the main exchange region of the placenta^4^,^28^. A relation to placental function is likely for modifications of tortuosity of branches in this region. All tortuosity values in position bT1 of the villous trees from IUGR placentas were below a threshold value of 1.2. In contrast, the tortuosity values in position bT1 of villous trees of 14 out of 50 (28%) investigated clinically normal placentas were substantially higher than this threshold (HT-normal; Fig. 2). Further detailed analysis revealed that the branches in position bT1 of the HT-normal group of the clinically normal placentas were on average longer and had on average a greater surface area and a larger mean volume of branches in positions bT0 and bT1 compared to branches of the LT-normal group of the clinically normal placentas (Fig. 4). They have thus properties which can be expected for structures that were formed to compensate for a possible deficit in maternofetal exchange (Fig. 5). In the IUGR group we could not detect a single case with such highly tortuous branches in bT1. Basically, this could be indicative of a higher flexibility of a subset of placentas to respond to rheologic challenges by adaptation of the structure of villous trees, resulting in clinically normal placentas with certain morphological alterations compared to other clinically normal placentas (outlined in detail in Figs 5D and 6C).

If this hypothesis is correct, the HT-normal group of placentas identified for the first time in the present study represents a subgroup of clinically normal placentas with successful compensation of rheologic disturbance. Without this structural adaptation of villous trees the development of symptomatic disease may be unavoidable (Figs 5D and 6B). Accordingly, for the first time, the present study points to a morphological correlate of compensated “ischemic placental disease^18^,^19^,^20^,^21^,^22^,^29^”. That placentas could be able to evade the rheologic disturbance caused by insufficient remodeling of uterine spiral arteries is a novel aspect. This view is not only important for basic research^18^,^19^,^20^,^21^,^22^,^29^ and prenatal programming, but if confirmed in future studies will open a totally new paradigm which could in the future be therapeutically instrumentalized to minimize the fraction of patients with ischemic placental disease which becomes clinically symptomatic.

However, it must be kept in mind that many aspects remain open at this stage. For instance, we do not know whether the degree of remodeling of uterine spiral arteries and severity of rheologic disturbance have to be below a critical threshold to allow compensation by the villous tree. Another option can be that certain placentas are intrinsically (by genetic or other reasons) not able to adapt to the modified blood flow in the intervillous space.

The clinical and macroscopic findings of the present study were in line with earlier findings in the literature^1^,^4^, demonstrating that placentas of pregnancies with IUGR were statistically significantly smaller and younger than clinically normal placentas (Table S1). The feto-placental weight ratio and the roundness of the outer rim of IUGR placentas were not significantly different statistically between IUGR placentas and clinically normal placentas (Table S1). This can be interpreted such that the size of the placenta and fetal weight are strictly coupled to each other^1^,^4^, irrespective of the difference of gestational ages between IUGR placentas and clinically normal placentas in the present study and the presence or absence of symptomatic obstetric disease. The hypothesis that variability of placental form might be associated with IUGR^30^,^31^,^32^,^33^ is not supported by the data of the present study.

Finally it should be mentioned that the novel 3D light microscopic method used in the present study has some limitations. Although the novel 3D microscopic analysis provides unique access to branching topology (including branching angles, length, tortuosity and nodes), the analysis of villous stroma and angiogenesis is not possible because no histological sectioning is involved. The latter also leads to the limitation that only the most peripheral branches of the placental villous tree can be examined, whereas data on larger stem villi and anchoring villi cannot be obtained because they cannot be investigated at the light microscopic level without histological sectioning.

## Conclusion

The 3D microscopic architecture of villous trees of IUGR placentas and clinically normal placentas differs in two main aspects: (i) the branching angles of branches at terminal positions bT0 are on average larger in IUGR, and (ii) villous trees of IUGR placentas do not show a remarkable population of longer and more tortuous villi in the directly preterminal part (bT1) of the villous trees of clinically normal placentas. The shift to larger angles in position bT0 can be indicative of rheologic disturbance caused by insufficient remodeling of uterine spiral arteries. The highly tortuous population of villous branches in position bT1 in a subgroup of clinically normal placentas can be interpreted as morphological correlate of functional compensation of insufficient uterine spiral arterial remodeling. In this subgroup of clinically normal placentas, symptomatic development of IUGR could thus possibly be avoided though signs of rheologic disturbances (indicated by higher branching angles in position bT0) were present.

## Materials and Methods

### Study design

The present study used two collections of placental tissue. We included 40 placentas from patients with intrauterine growth retardation (IUGR placentas) and 50 placentas from clinically normal pregnancies (clinically normal placentas). Both cohorts of placentas were collected at the Department of Obstetrics and Gynecology of the hospital “Dritter Orden”, Munich, Germany. The collection of clinically normal villous trees was used in a previous study^13^ and served as control group for the collection of IUGR placentas. The course of each pregnancy was assessed by obstetricians based on clinical information regarding the pregnancy and delivery. An IUGR was diagnosed if the growth data of the fetus determined by ultrasound (e.g. femur length, abdomen and head circumferences, etc.) were above the 10th growth percentile during the first two trimesters and then dropped below the 10th growth percentile. Patients with a combination of IUGR and symptoms of preeclampsia were not included.

Placentas were collected after informed consent of the mothers/parents was obtained. Placentas were excluded when (i) no informed consent of the mothers/parents could be obtained, (ii) the language skills of the mothers/parents limited the understanding of information concerning the study, or (iii) psychiatric problems or any other condition caused doubts regarding the mothers/parents ability to independently decide. All work was conducted according to relevant guidelines and regulations. This study was approved by the ethics committee of the Ludwig-Maximilians-University of Munich (Munich, Germany) under the numbers **084-11** and **478-12**. All data were anonymized. The thickness of the placentas was determined by ultrasound. The placental weight (PW) was measured prior to tissue sampling and processing without the umbilical cord but with membranes^1^,^4^,^34^. The birth weight (BW) was determined immediately after birth, and the feto-placental weight ratio (PW/BW) was calculated (Supplementary Table S1). The cohort of clinically normal placentas was collected between February 2012 and April 2013^13^, and the cohort of IUGR placentas between January 2013 and August 2014.

### Procedure of tissue sampling and preparation

All placentas were cooled at 4 °C immediately after birth and processed for histology at the Department of Anatomy II of Ludwig-Maximilians-University (Munich, Germany) as described earlier^13^.

For the preparation of single, isolated peripheral parts of villous trees, a sample (edge length, 2–3 cm) was collected. The sampling site was determined using a systematic and random sampling scheme based on the projection of a point pattern onto the chorionic surface of the placenta^30^. Six sampling sites were taken for routine paraffin embedding and histology; the sampling of the isolated peripheral villous trees was done from the remaining unfixed placental tissue at the midpoint between sampling sites 3 and 4^30^, a spot that turned out to be not more than 5 cm distant from the insertion of the umbilical cord in all cases. The sample was excised and transferred to physiological saline at 4 °C as previously described^13^. The preparation of peripheral villous trees began within one hour after sampling for histology. Free bushes of peripheral villi were identified, removed using small scissors, and fixed in 4.5% formaldehyde. After washing and bleaching the villous trees were stained with Mayer’s hematoxylin. After dehydration in a graded series of ethanol, the probes were mounted in DPX on a concave slide. These preparations have a true 3D character and are free from oppressing contact with the concave slide or cover glass^13^.

### 3D analysis of villous trees

**Figure 7:**
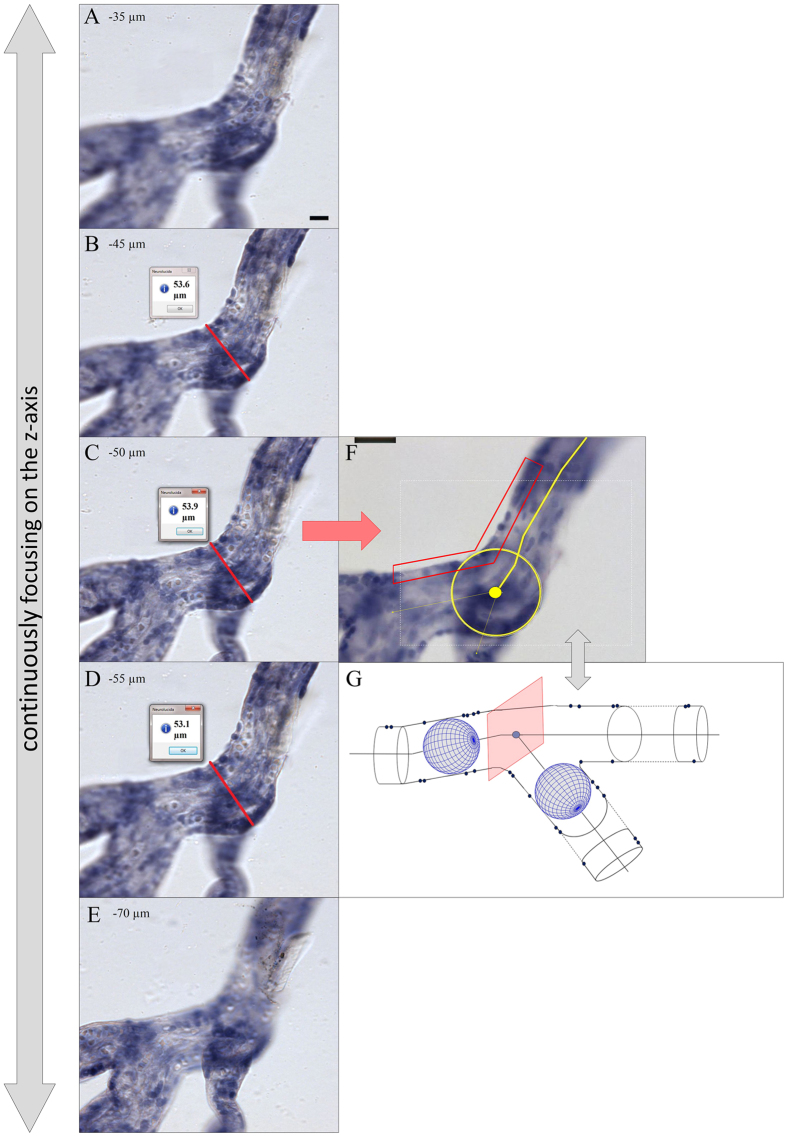
Identification of center lines and branching points of human placental villous segments with 3D light microscopy. (**A-E**) Selected screenshots of the same microscopic field-of-view taken at various focus depths during an analysis session of a villous tree using Neurolucida software (MBF Bioscience) through a distance of 35 μm in Z direction (starting at −35 μm in A through −70 μm in E). The procedure of positioning the center point at a node is illustrated. The center point was positioned at the Z position at which the investigated villous segment had its maximum diameter (found in panel C; red lines in panels B-D represent the “quick measure line” function of the Neurolucida software). At a focus depth of −50 μm (panel C) the largest diameter of the investigated villous segment was in focus. (**F,G**) illustrates how the center point of the investigated villous segment at the shown XY position was identified with the Neurolucida software. At the Z position with the largest diameter in focus (panel C) a circle was opened which defined the center point (red arrow from C to F). Note that the marginal cell nuclei of trophoblast were in focus at this Z position (red rectangular label in F) which was used as additional criterion for correct positioning of the center point at the selected Z position. (**G**) Neurolucida’s approach is comparable to software algorithms of 3D analysis which are based on “virtual rolling balls” to define center lines and branching points. The scale bar in (A) represents 25 μm in (**A-E**), and the scale bar in (**F**) 25 μm in (**F**).

All peripheral villous trees were traced with Neurolucida^35^,^36^,^37^ (version 10.54; MBF Bioscience) under a light microscope using a 20x objective lens with the working direction from the proximal toward the terminal end of the peripheral villous tree as previously described (Fig. 1)^13^. The diameter of branches was determined continuously as a frustum around the center point. To position the center point correctly in 3D, the diameter was determined in the focus plane showing the largest diameter of the branch. In case of doubt, the “quick measure line” function of the software Neurolucida was used to determine the correct focus plane (Fig. 7). In addition, the trophoblast nuclei of the branch surface should be visible at the margin (Fig. 7) in the same plane. Using these two criteria, the center points were correctly positioned in 3D and the center line connecting them provided an accurate 3D skeleton for determination of branching points and branching angles (Fig. 1). Tree ordering for measurements was set to “Terminal Distance Ordering” in Neurolucida because the terminal end was the biologically defined end of the isolated peripheral villous trees (Fig. 1). Terminal Distance Ordering classifies branches according to their distance in nodes from the terminal end of the villous tree. The measuring system generated a digital 3D replica of the peripheral villous tree under investigation in parallel to the process of tracing (Fig. 1). These data were analyzed with Neurolucida Explorer software (MBF Bioscience) using the option “branching structure analysis”.

We used two microscope systems: (i) an Axioskop (Zeiss, Goettingen, Germany) with a motorized XYZ specimen stage (Maerzhaeuser, Wetzlar, Germany), LEP MAC6000 XYZ 3-axis stage controller (Ludl), focus encoder (Type MT 1271; Heidenhain), and color digital camera (3/40 CCD chip 1,92 MP, 1600×1200 pixel, MBF Bioscience; and (ii) a BX50 (Olympus, Tokyo, Japan) with motorized XYZ specimen stage (MBF Bioscience), LEP MAC6000 XYZ 3-axis stage controller (Ludl), focus encoder (Type MT 1271; Heidenhain) and color digital camera (1/20 CCD chip, 1392 × 1040 pixel, MBF Bioscience). The parts of villi connecting two nodes or connecting a terminal end with a node were named branches (b). Branches were further classified by their distance to the nearest terminal end (bT, with the T indicating classification by terminal distance). The distance was measured by the number of nodes to the nearest terminal end, i.e., bT0 (terminal end), bT1 (one node apart of the next terminal end) and bT2 (two nodes apart of the next terminal end^13^.

Furthermore, we collected data regarding the planar branching angle, diameter, length, surface area and volume of each individual branch. These data were aggregated by the terminal distances of branches for each villous tree (Fig. 1). Of 50 peripheral villous trees of clinically normal placentas, 11 did not show bT2 branches. Of the remaining 39 bT2 branches the planar branching angle of bT2 branches could not be determined for six samples due to a missing previous branch (n = 33 for mpa bT2)^13^. Of the 40 peripheral villous trees of IUGR placentas, 18 did not show bT2 branches (n = 22 for bT2).

### Statistical analysis

For all investigated parameters, the mean and standard deviation (SD) were calculated using SPSS software (Version 23; IBM, Armonk, NY, USA)^38^. Student’s t-test and Kruskal-Wallis test with Dunn’s comparison test^38^ were performed with GraphPad Prism software (Version 5; GraphPad, San Diego, CA, USA). A post-hoc statistical analysis was carried out to explore properties of two subpopulations selected from clinically normal villous trees. The two subpopulations distinguished one group with high tortuosity in bT1 (HT-normal) and another group with low tortuosity in bT1 (LT-normal). Rose diagrams of the data on branching angles were prepared with the function rose.diag using the software R^39^ and the R package CircStats^40^. Unimodal/multimodal distribution of the data on branching angles was tested with Hartigan’s Dip Test^41^ using the software R^39^.

## Additional Information

**How to cite this article**: Haeussner, E. *et al*. Novel 3D light microscopic analysis of IUGR placentas points to a morphological correlate of compensated ischemic placental disease in humans. *Sci. Rep.* **6**, 24004; doi: 10.1038/srep24004 (2016).

## Supplementary Material

Supplementary Information
