# Supplementary material for: Novel 3D light microscopic analysis of IUGR placentas points to a morphological correlate of compensated ischemic placental disease in humans
Source: Sci Rep. 2016 Apr 5;6:24004. doi: 10.1038/srep24004 (PMC4820778; doi:10.1038/srep24004)
Supplement: Supplementary Information [file srep24004-s1.doc]

**Novel 3D light microscopic analysis of IUGR placentas points to a morphological correlate of compensated ischemic placental disease in humans**

Eva Haeussner, Christoph Schmitz, Hans-Georg Frank, Franz Edler von Koch

**Supplementary Information**

Table S1 Descriptive statistic of macroscopic parameters (gestational age (GA), birth weight (BW), placental weight (PW), feto-placental weight ratio (PW/BW), surface area (surface), roundness of placental disc (roundness), thickness of placental disc (thickness), longest diameter of placental disc (LD), shortest diameter of placental disc (SD)) of intrauterine growth restriction (IUGR) and clinically normal placentas is shown. The number (n), mean and standard deviation (SD) is given per case. The p-value of the Student’s t-test between IUGR and clinically normal placentas is given in the last column.

| **IUGR** | **n** | **mean** | **SD** | **clinically normal** | **n** | **mean** | **SD** | **t-Test p-value** |
| --- | --- | --- | --- | --- | --- | --- | --- | --- |
| GA (weeks) | 40 | 37.53 | 2.72 | GA (weeks) | 50 | 39.29 | 1.03 | **p < 0.001** |
| BW (g) | 40 | 2273.10 | 498.96 | BW (g) | 50 | 3456.98 | 583.09 | **p < 0.001** |
| PW (g) | 40 | 350.40 | 85.66 | PW (g) | 50 | 524.06 | 121.69 | **p < 0.001** |
| PW/BW ratio | 40 | 0.160 | 0.049 | PW/BW ratio | 50 | 0.150 | 0.023 | **ns** |
| surface area (cm²) | 40 | 915.50 | 244.43 | surface area (cm²) | 50 | 1143.70 | 243.43 | **p < 0.001** |
| roundness | 40 | 1.21 | 0.15 | roundness | 50 | 1.19 | 0.16 | **ns** |
| thickness (cm) | 40 | 1.51 | 0.35 | thickness (cm) | 50 | 1.75 | 0.37 | **p < 0.01** |
| LD (cm) | 40 | 18.54 | 2.56 | LD (cm) | 50 | 20.71 | 2.83 | **p < 0.001** |
| SD (cm) | 40 | 15.50 | 2.41 | SD (cm) | 50 | 17.45 | 1.97 | **p < 0.001** |


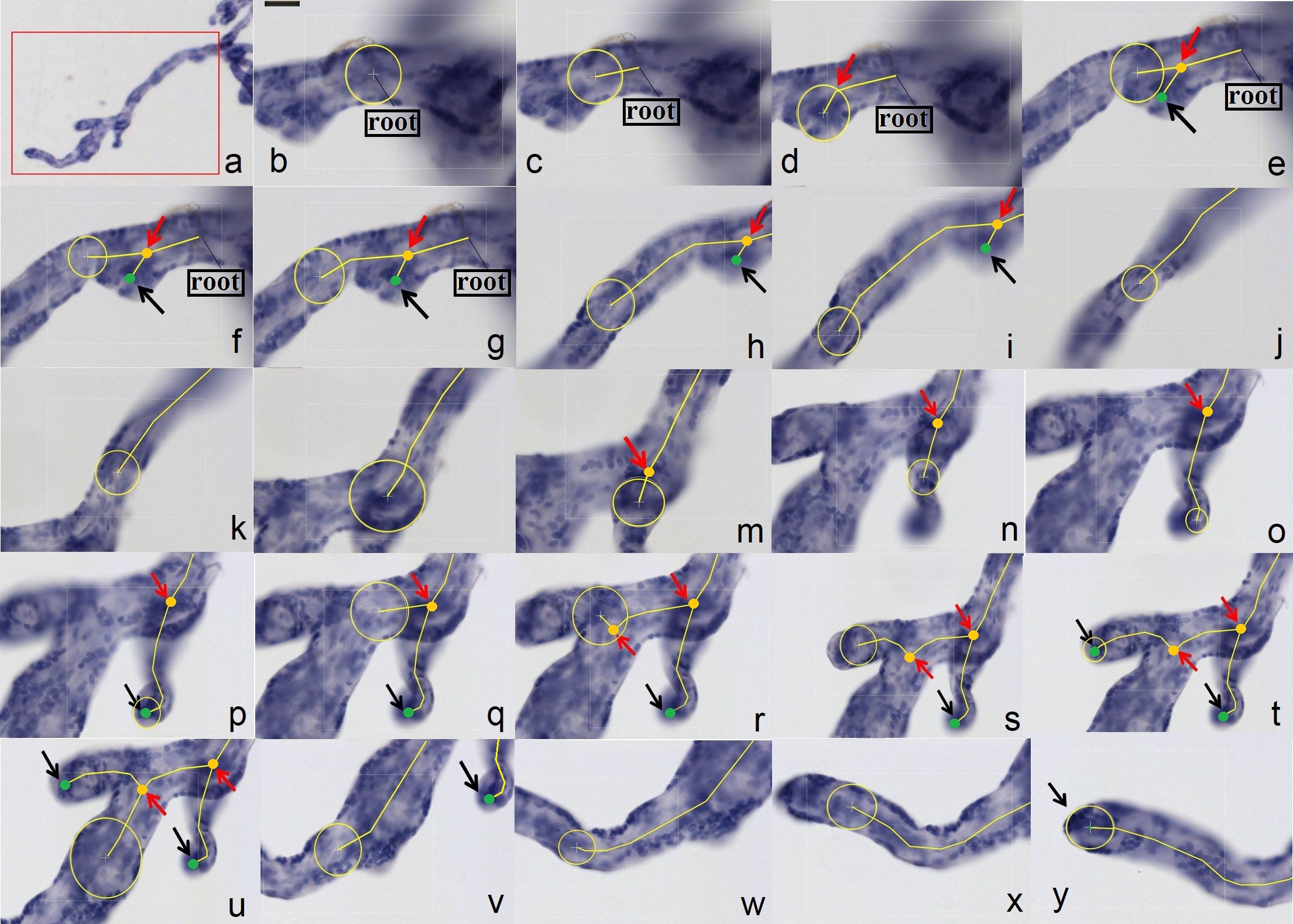


Figure S1 Computer-assisted viewing generates a quantitative digital three-dimensional (3D) replica of villous trees of the human placenta. (a–y) Example illustrating the procedure by which the Neurolucida software assisted in generating a digital and quantitative 3D replica of a whole-mount preparation of an isolated villous tree of a human placenta. The entire instrument and software setup was calibrated such that the software recorded 3Dcoordinates of all positions and mouse functions. Measurement and 3D-reconstruction were simultaneous processes. (a) shows an overview at low magnification (2x objective). The red box in (a) delineates the region of interest. The region of interest is larger than a single microscopic field-of-view at working magnification (20x objective). (b–y) Screenshots taken at working magnification (20x objective; scale bar in (b) 25 µm on the top is equal for each tile) while the tracing of the villous tree in the region of interest was in progress. The coordinates of the centerpoint of the yellow circle and the diameter of this circle were recorded while the villous tree was traced in x,y, and z. The work flow started with the most proximal point of the region of interest (root point of the model, (b)). The yellow circle shown could be adjusted to the diameter of the villus in focus using the mouse wheel function. Its moving center defined the center line (yellow line) of each branch. Branching points (nodes; shown as yellow dots with associated red arrows) were placed by clicking functions. Similarly, terminal ends were defined using the mouse wheel function and are shown as green dots with associated black arrows (copy of reference 13 with permission).
